# CRISPR/Cas9: A Powerful Strategy to Improve CAR-T Cell Persistence

**DOI:** 10.3390/ijms241512317

**Published:** 2023-08-01

**Authors:** Wei Wei, Zhi-Nan Chen, Ke Wang

**Affiliations:** National Translational Science Center for Molecular Medicine & Department of Cell Biology, Fourth Military Medical University, Xi’an 710032, China; weiweicell@163.com

**Keywords:** CAR-T cell, CRISPR/Cas9, persistence, memory phenotype, exhaustion

## Abstract

As an emerging treatment strategy for malignant tumors, chimeric antigen receptor T (CAR-T) cell therapy has been widely used in clinical practice, and its efficacy has been markedly improved in the past decade. However, the clinical effect of CAR-T therapy is not so satisfying, especially in solid tumors. Even in hematologic malignancies, a proportion of patients eventually relapse after receiving CAR-T cell infusions, owing to the poor expansion and persistence of CAR-T cells. Recently, CRISPR/Cas9 technology has provided an effective approach to promoting the proliferation and persistence of CAR-T cells in the body. This technology has been utilized in CAR-T cells to generate a memory phenotype, reduce exhaustion, and screen new targets to improve the anti-tumor potential. In this review, we aim to describe the major causes limiting the persistence of CAR-T cells in patients and discuss the application of CRISPR/Cas9 in promoting CAR-T cell persistence and its anti-tumor function. Finally, we investigate clinical trials for CRISPR/Cas9-engineered CAR-T cells for the treatment of cancer.

## 1. Introduction

In the past decade, immunotherapy has achieved remarkable results in the treatment of malignant tumors, including immunosuppressive checkpoint blockade, non-specific immunomodulators, adoptive cell therapy, and tumor vaccines. Among these therapeutic options, adoptive cell therapy (ACT) is the transfer of immune cells, especially T lymphocytes, with robust anticancer activity into patients. Since its inception, ACT has exhibited prominent effects in its clinical application and has become a promising therapeutic approach to cancer. As one type of ACT treatment, chimeric antigen receptor T (CAR-T) cells are genetically engineered T cells that express a chimeric antigen receptor so as to recognize and target specific cancer cells [1]. To date, eight CAR-T cell products against CD19 or BCMA have been approved for use in clinical practice. CD19-CART cells are used to treat B-cell leukemias, while BCMA-CART cells have been approved to treat multiple myeloma (MM). According to the results of reported clinical trials, over 80% complete remission was acquired in refractory or relapsed B-cell acute lymphoblastic leukemia (r/r B-ALL) patients after CD19-CART cell treatment [2], and 85% complete remission was reached in acute lymphoblastic leukemia (ALL) patients [3]. Meanwhile, CD19-CART cells have been approved as a second-line treatment for chemotherapy-failed large B-cell lymphoma patients [4]. For BCMA-CART cells, a 67% complete remission was achieved in patients with relapsed/refractory multiple myeloma [5,6]. The clinical trial results have demonstrated that CAR-T cell therapy is a promising treatment in hematological malignancies and is worth being promoted in clinical applications. Nevertheless, no CAR-T cell product has been approved for use in the clinic to treat solid cancers. Currently, clinical trials of CAR-T cells against solid tumors are ongoing, including against breast cancer [7,8], glioblastoma [9,10,11], liver cancer [12], prostate cancer [13], lung cancer [14], and gastrointestinal cancers [15]. Several clinical trials exhibited that effective tumor control was initially achieved after CAR-T cell treatment, but long-term tumor regression cannot be sustained [16]. The absence of tumor-specific antigens, limited infiltration of CAR-T cells into tumor sites, antigen loss, immunosuppressive tumor microenvironment (TME), and poor persistence of CAR-T cells are major factors that limit the efficiency of CAR-T cell therapy in solid tumors. Meanwhile, the hypofunction of autologous T cells derived from tumor patients also restrains the efficiency of CAR-T cells. Thus, it is urgent to improve the therapeutic effect of CAR-T cells in solid tumors. Meanwhile, although CD19-CART cells have achieved prominent clinical success in the treatment of relapsed/refractory B-cell hematological malignancies in children and adults, it has been demonstrated that about 40–60% of patients eventually recrudesce after a period of time [17]. It has been well recognized that the limited persistence and survival of CAR-T cells contribute to the relapse. Accumulating evidence shows that the long-term persistence of CAR-T cells is positively associated with a durable anti-tumor response [18,19,20]. Several factors hinder the long-term persistence of CAR-T cells in vivo, including the differentiation phenotype, exhaustion status, and CAR structure [21,22]. However, genetic modifications can be applied to engineer CAR-T cells to overcome these obstacles, thus improving their persistence and survival in vivo. 

Genome editing is a type of genetic engineering in which DNA is modified, deleted, replaced, or inserted into the genome of cells [23,24]. To date, various gene-editing tools have been applied for engineering various genomes, such as zinc finger nucleases (ZFNs), transcriptional activator-like effector nucleases (TALENs), and the clustered regularly interspaced short palindromic repeat (CRISPR)/CRISPR-associated nuclease 9 (Cas9) system [25]. Unlike ZFNs or TALENs, which use proteins to target DNA sites, CRISPR/Cas9 directs Cas proteins to the specific DNA site under the guidance of a single RNA whose sequence is complementary to the targeted DNA [26]. Compared to ZFNs and TALENs, the CRISPR/Cas9 system has a higher accuracy and efficiency. Owing to its simple design, easy use, low cost, and high efficiency, CRISPR/Cas9 technology has been widely used in biological studies [27,28]. In the context of CAR-T cell therapy, CRISPR/Cas9 technology has been used to prolong CAR-T cell persistence, reverse exhaustion, and enhance anti-tumor efficacy [29]. Meanwhile, CRISPR/Cas9 has been applied in clinical trials to engineer CAR-T cells [30]. In this review, we summarize the applications of the CRISPR/Cas9 system in CAR-T cells and its clinical status, with the hopes of offering an innovative strategy to improve CAR-T cell function and promoting the durable clinical remission of tumor patients.

## 2. Factors Limiting CAR-T Cell Persistence and Clinical Response

Although CAR-T cell therapy has achieved remarkable clinical responses in hematological malignances, 40-60% of patients eventually relapse after CAR-T cell treatment. It has been demonstrated that CAR-T cells were undetectable in the peripheral blood of relapsed patients [31]. Increasing clinical experience has indicated that the clinical efficiency of CAR-T cell therapy is positively associated with in vivo proliferation and persistence [18,32,33]. The vigorous expansion and long-term persistence of CAR-T cells in vivo could maintain a durable clinical remission. It has been suggested that the phenotypes of infused CAR-T cells and CAR-T cell exhaustion are two important determinants that impair the long-term persistence of CAR-T cells [34,35].

### 2.1. CAR-T Cell Differentiation Phenotype Determines Anti-Tumor Potency

Pre-clinical and clinical experience have demonstrated that the differentiation status of T cells is closely bound up with the anti-tumor ability and durability of T cells [36,37,38,39]. According to the differentiation stage, T cells can be subdivided into naïve T (T_N_) cells, stem cell memory T (T_SCM_) cells, central memory T (T_CM_) cells, effector memory T (T_EM_) cells, and terminally differentiated effector T (T_EFF_) cells [40]. Immature T cells are considered T_N_ cells before they are encountered with their homologous antigens. Naïve T cells highly express CD45RA isoform, L-selectin (CD62L), and CXC chemokine receptor 7 (CCR7) [41]. The last two molecules play a leading role in guiding T lymphocytes homing to the secondary lymphoid organs. Naïve T subsets possess high self-renewal and survival capacity [42]. After interacting with cognate antigens, T_N_ cells are stimulated to expand and differentiate into memory cells and/or effector T cells [43]. As the less differentiated subsets among memory T cells, stem cell memory T (T_SCM_) cells have attracted much attention in adoptive cell therapy. With the properties of T_N_ and T_M_ cells, this subset exhibits robust survival potency in vivo. T_SCM_ cells respond more quickly to antigen stimulation and survive longer than other memory T cells [44,45]. The markers expressed on T_SCM_ cells include CD45RA, CD62L, CCR7, and CD95. Central memory T (T_CM_) cells express CD45RO, CD62L, CCR7, and CD27, but not CD45RA, and could home to second lymphoid organs [46]. Although T_CM_ cells are less cytotoxic than T_EFF_ cells, their stronger proliferation and survival potency contribute to a more effective anticancer response [47]. As a result, the proportion of T_SCM_ and T_CM_ cells has become an effective index for evaluating the efficacy of CAR-T cell therapy. Due to the deficiency of CD62L and CCR7 expression, effector memory T (T_EM_) cells cannot circulate to lymph nodes, while they can access target tissues and are more cytotoxic than T_CM_ cells [48]. Under persistent antigen stimulation, T_EM_ cells eventually differentiate into terminal effector T (T_EFF_) cells. Like T_EM_ cells, this subset also does not express CD62L and CCR7. T_EFF_ cells play the major role in lysing tumor cells but have poor self-renewal ability and limited persistence in vivo. Meanwhile, effector T cells are more vulnerable to becoming exhausted than memory T cells [49]. Accordingly, the conventional CAR-T cell products composed mainly of effector T cells show limited persistence after infusion [50]. Compared with T_EM_ and T_EFF_ cells, less differentiated T cells, including T_N_, T_SCM_, and T_CM_ cells, exhibit vigorous expansion and persistence in vivo. T_EFF_ subsets show lower proliferation and are more likely to be exhausted and reduced homing to tumor sites than memory T cells. CAR-T cells generated from T_N_, T_SCM_, and T_CM_ cells have exhibited greater anti-tumor ability and long-term persistence in preclinical studies [50,51,52,53]. Hence, it is necessary to induce more less differentiated subsets in CAR-T cells so as to promote long-time persistence and improve anti-tumor capacity.

### 2.2. CAR-T Cell Exhaustion Impairs Long-Term Anti-Tumor Activity

The exhaustion of T cells is first reported in chronic lymphocytic choriomeningitis virus (LCMV) infection [54] and later in multifarious tumors and autoimmune diseases [55]. During an acute infection, naïve T cells are activated and differentiated into cytotoxic effector T cells to eliminate enthetic pathogens or antigens. Once the pathogens or antigens are cleared, the majority of cytotoxic T cells evolute toward apoptosis, while a minority differentiate into memory T cells, persisting in the body to protect against the second infection. However, in chronic virus infections or tumors, T cells are persistently stimulated by antigens. Under sustaining antigen exposure, T cells are transformed into exhausted T cells. T-cell exhaustion is a status of dysfunction and hyporesponse, which manifests as impaired anti-tumor ability, reduced cytotoxicity and effector cytokine release, such as GranzymeB and IFN-γ, attenuated proliferation potential, and persistent expression of checkpoint molecules like programmed cell death protein 1 (PD-1), cytotoxic T-lymphocyte-associated antigen 4 (CTLA-4), T-cell immunoglobulin domain and mucin domain-3 (TIM3), and lymphocyte-activation gene 3 (LAG3) [56]. The exhausted T cells are heterogeneous in phenotype and function and can be divided into progenitor exhausted T (T-bet^high^ Eomes^low^ PD-1^int^) and terminally exhausted T (T-bet^low^ Eomes^high^ PD-1^high^) subsets [57]. The progenitor exhausted T cells possess a high self-renewal ability and contribute to the effective response to PD-1 blockade. While terminally exhausted T cells lose their proliferation potency and are unable to respond to PD-1 checkpoint blockade therapy. Under consistent antigen exposure, effector T cells would undergo a series of transcriptional regulation and epigenetic remodeling. Thymocyte selection-associated high mobility group (HMG) box protein TOX, nuclear receptor NR4A, and nuclear factor of activated T cell (NFAT) are crucial regulators for the development of exhausted T cells [58]. Due to the impaired cytotoxicity against tumor cells and attenuated proliferation potential, the exhausted T cells cannot effectively exert anti-tumor ability and sustain persistent tumor control potency. Thus, T-cell exhaustion has been a major contributor to the failure of adoptive cell therapy in tumors. Several clinical studies have demonstrated that T-cell exhaustion is correlated with the treatment failure in B-ALL and B-cell lymphoma patients after CAR-T cell administration and CAR-T cells from non-responders are highly exhausted [34,59]. Hence, reversing T-cell exhaustion will be a promising strategy to improve the efficiency of CAR-T cell therapy.

Despite the fact that the activation mechanisms of CAR are distinct from the T-cell receptor (TCR), exhaustion is also discovered in CAR-T cells and the degree of T-cell exhaustion determines the therapeutic efficacy of CAR-T cells. In conventional CAR-T cell products, most are effector T cells, which are easily exhausted and lose their cytotoxic ability. Due to CAR-T cell exhaustion, the clinical responses are attenuated, eventually leading to tumor relapse [56]. The mechanisms of CAR-T cell exhaustion have not been fully understood, but several factors that induce the exhaustion of CAR-T cells have been elucidated. The CAR structure and in vitro culture condition can lead to the exhaustion of CAR-T cells [60]. For instance, CAR-T cells possessing the CD28 co-stimulation domain are more prone to be exhausted, while the 4-1BB co-stimulation domain could ameliorate T-cell exhaustion [61]. For in vitro expansion systems, IL-2 could induce CAR-T cell exhaustion and inhibit in vivo persistence when added to the culture system [62]. In addition, CAR-T cells are more prone to be exhausted in solid tumors due to the immunosuppressive TME [16]. Immunosuppressive cells and inhibitory cytokines play major roles in inducing CAR-T cell exhaustion in solid tumors. Immunosuppressive cells, including myeloid-derived suppressor cells (MDSCs), cancer-associated fibroblasts (CAFs), regulatory T (Treg) cells, and tumor-associated macrophages (TAMs) can directly or indirectly induce the exhaustion of T cells [63]. For MDSCs, TAMs, and CAFs, they would upregulate the expression of immune checkpoint molecules, like PD-L1 and CTLA4, which can induce CAR-T cell exhaustion after binding to their ligands on T cells [64,65,66]. Meanwhile, these immunosuppressive cells also secrete suppressive cytokines such as IL-10 and TGF-β to inhibit the CAR-T cell effector function. In addition, MDSCs and TAMs could deplete some nutrients that are essential for T-cell functions [64,65]. Treg cells can inhibit effector T-cell activation and expansion, increase inhibitory receptors’ expression on effector T cells, and release suppressive cytokines like IL-10 and TGF-β [67,68]. IL-10 could inhibit CD8^+^ cytotoxic T-cell activation and proliferation [69,70], while TGF-β could promote Treg cell differentiation [71], upregulate PD-1 expression on T cells [72], and induce the apoptosis of CD8^+^ cytotoxic T cells [73]. Other pro-tumor cytokines such as IL-6 and chemokines (such as CCL2, CCL5, CCL22, CXCL1, and CXCL2) secreted from immunosuppressive cells also exert an inhibitory function on T cells [70,74]. The engineering CAR-T cells with enhanced resistance to the immunosuppressive microenvironment can effectively avoid exhaustion, thereby improving their anti-tumor therapeutic effects.

## 3. CRISPR/Cas9 Application in Improving the Persistence of CAR-T Cells

As a gene-editing tool with high efficiency and precision, CRISPR/Cas9 has acquired wide applications. This system comprises the CRISPR-associated protein Cas9 and a single-guided RNA (sgRNA). The Cas9 protein is a DNA endonuclease enzyme that can create double-stranded breaks (DSBs) in the target DNA sites. The sgRNA is a ~20 nucleotide sequence that is complementary to the targeted DNA, which can direct the Cas9 protein to target DNA cut sites [75]. sgRNA possesses a protospacer adjacent motif (PAM) sequence at the 3′ end of its sequence, which is essential for the initial recognition and disruption of the target DNA by sgRNA-Cas9 [76]. After the binding of the sgRNA with the target DNA, the Cas9 protein produces a DSB from three nucleotides upstream of the PAM sequence. The CRISPR system was first demonstrated as an adoptive immune system in prokaryotes to defend against phage infections and plasmid transfer [23,77]. Since then, CRISPR/Cas9 has been investigated in eukaryotes to create site-specific gene edits. Several distinct technologies have been utilized for delivering the CRISPR/Cas9 system into cells [78]. The first one is to utilize the same vector-carrying plasmid DNA encoding the Cas9 protein and sgRNA. The second approach is the combination of sgRNA and Cas9 mRNA. Another format is a ribonucleoprotein (RNP), comprising Cas9 protein and sgRNA, exhibiting more efficient and safer than the other two strategies. Lentiviral and adenoviral vectors for delivering the CRISPR system into primary T cells exhibit limited efficiency. While the RNP delivery strategy is an effective approach as it can be electroporated into T cells. As an efficient gene-editing tool, the CRISPR/Cas9 system has markedly promoted the anti-tumor function of CAR-T cells in preclinical and clinical investigations.

### 3.1. Disruption of Immune Checkpoints in CAR-T Cells

T-cell exhaustion is a state of dysfunction and hyporesponse, which manifests as impaired anti-tumor ability and attenuated proliferation potential. One hallmark of T-cell exhaustion is the persistent expression of inhibitory receptors, including PD-1, CTLA-4, LAG3, and TIM3. Upon binding to their ligands, inhibitory signals are transmitted into T cells and induce cell exhaustion [79,80]. PD-1 and CTLA4 are pivotal immune checkpoints in T-cell exhaustion [81]. PD-1 is the hallmark of T-cell exhaustion and functions in the late stages of T-cell activation. After binding to its ligand PD-L1/PD-L2, it inhibits T-cell activation and decreases the release of cytotoxic cytokines like IFN-γ, IL-2, and TNF-α [82]. CTLA-4 is a member of the immunoglobulin superfamily-related receptors and has a higher affinity than CD28 binding to B7 on antigen-presenting cells, thus leading to the exhaustion of T cells [83]. Meanwhile, CTLA-4 is also expressed on Tregs and can enhance the immunosuppressive function of Tregs [84]. The concrete mechanisms of TIM3 and LAG3 in T-cell exhaustion are not entirely elucidated, but these inhibitory receptors are co-expressed with PD-1 and play important roles in T-cell exhaustion [70]. Clinical trials have demonstrated that immune checkpoint blockades could significantly reduce T-cell exhaustion and prolong the survival of tumor patients [85]. For CAR-T cells, persistent tumor antigen stimulation and immunosuppressive signals in TME concurrently induce their exhaustion. The exhausted CAR-T cells lose their cytotoxic and long-term survival abilities. The disruption of inhibitory receptors by CRISPR/Csa9 technology in CAR-T cells can significantly enhance its resistance to the immunosuppressive TME and is a promising immunotherapy modality for cancer treatment. Several studies have investigated the disruption of immune checkpoints by CRISPR/Cas9 in CAR-T cells. PD-1-disrupted EGFRvIII-CART cells exhibit enhanced cytotoxicity and reduced exhaustion in glioblastoma [86,87]. Likely, PD-1 disruption in mesothelin-targeted CART cells strongly augments cytokine production, enhances tumor control, and prevents relapse in breast carcinoma [88]. Also, PD-1-deleted GPC3-CART cells show enhanced in vivo anti-tumor activity against HCC without impairing GPC3-CART cell activation and proliferation [89]. For fluid tumors, PD-1-knockout CD19-CART cells are continuously exposed to antigens and survive over 390 days in a syngeneic immunocompetent mouse model [90]. All the evidence demonstrates that CRISPR/Cas9-mediated PD-1 disruption could effectively suppress CAR-T cell exhaustion and prolong CAR-T cell persistence in vivo. Besides, LAG3 knockout-CART cells exhibit vigorous anti-tumor ability in vitro and in a mouse model [91]. All the above results demonstrate that immune checkpoint disruption by CRISPR/Cas9 is a promising strategy to revitalize CAR-T cells.

### 3.2. The Deletion of Negative Regulators of CAR-T Cell Function

The anergy of CAR-T cells after infusion is also regulated by other negative regulators and signals. The disruption of negative regulators by CRISPR/Cas9 can also rescue the CAR-T cell effector function. As an E3 ubiquitin ligase, the expression of Cbl-b is increased in exhausted CD8^+^ tumor-infiltrating lymphocytes (TILs) and promotes the exhaustion of TILs. Disrupting the expression of Cbl-b inhibits CAR-T cell exhaustion and invigorates their effector function against tumor cells [92]. Likely, PTP1B expression is also increased in exhausted CD8^+^ T cells, and PTP1B can inhibit cytokine-induced JAK/STAT signaling activation, which is essential for T-cell immunity. Thus, the disruption of PTP1B by CRISPR/Cas9 could enhance the activity of CAR-T cells to kill solid tumors [93]. In addition, Charly R. et al. identify that SOX4 and ID3 are pivotal regulators in CAR-T cell exhaustion, and disrupting ID3 and SOX4 could enhance the anti-tumor immunity of CAR-T cells by restraining their dysfunction [94]. The nuclear receptor transcription factor NR4A is highly expressed in CD8^+^-exhausted T cells, and the knockout of NR4A in CAR-T cells inhibits tumor progression [95]. Meanwhile, exhausted T cells are metabolically reprogrammed, and manipulating metabolism has been proven to be an effective approach to improving T-cell resistance against inhibitory TME [96]. Diacylglycerol kinases (DGKs) are a class of enzymes catabolizing diacylglycerols (DAGs) to phosphatidic acid, and the activation of DGKs leads to the inhibition of TCR signals [97]. Jung et al. reveal that the deletion of DGKs augments the CAR-T cell effector function by enhancing TCR signaling and alleviating CAR-T cell exhaustion [98]. Adenosine is another crucial metabolic mediator of immunosuppression in TME [99]. After binding to the adenosine A2A receptor (A2AR), adenosine exerts the immunosuppressive function on effector T cells [100]. The CRISPR/Cas9-mediated gene knockout of A2AR renders CAR-T cell resistance to adenosine-mediated immunosuppression and improves the activity of the JAK-STAT signaling pathway, resulting in an enhanced anti-tumor efficacy [101].

### 3.3. Enhancing CAR-T Cell Resistance to Suppressive Cytokines

In TME, tumor cells and immunosuppressive cells secrete multiple cytokines to induce the dysfunction of CAR-T cells, including TGF-β, IL-6, and IL-10 [63,102]. For example, TGF-β plays a vital role in inhibiting the function of CD8^+^ cytotoxic T cells. TGF-β transmits signals via binding to type I and type II TGF-β receptors. The deletion of the TGF-β receptor II in CAR-T cells by the CRISPR/Cas9 system could prevent CAR-T cell exhaustion, and TGF-β receptor II knockout CAR-T cells exhibit robust tumor killing activity [103]. In addition, another study shows that knockout of the TGF-β receptor II could also increase the amount of central memory and effector memory subsets within circulating CAR-T cells, resulting in long-term efficacy against solid tumors [104]. Cytokine-inducible SH2-containing protein (CISH) belongs to the suppressor of the cytokine signaling (SOCS) protein family [105] and can inhibit TCR signaling in CD8^+^ T cells [106], which is crucial for the activation and immune activity of T cells. The ablation of CISH prolongs the survival and improves the cytokine release and anti-tumor potency of CAR-T cells. Meanwhile, CISH deficiency decreases PD-1 expression on CAR-T cells, preventing the exhaustion of CAR-T cells [107]. In addition, cytokine release syndrome (CRS) and neurotoxicity limit the efficacy of CAR-T cells in clinical applications. The development of CRS has a direct correlation with the massive production of T-cell effector cytokines, such as GM-CSF [108]. The serum level of GM-CSF is closely related to the development of neurotoxicity [109]. Thus, regulating the secretion of GM-CSF in CAR-T cells will be a potential tactic to prevent CRS and neurotoxicity, thus enhancing the efficiency of CAR-T cells. It is demonstrated that GM-CSF-deficient CD19-CART cells by CRISPR/Cas9 display prolonged survival and enhance anti-tumor activity in vivo [110].

### 3.4. Epigenetic Reprogramming Strategies to Augment CAR-T Cell Effector Functions

Advances in high-throughput technologies reveal the important role of epigenetic remodeling in T-cell differentiation and function. Epigenetic regulation could alter the fate of T-cell differentiation and boost effector function. TET2 encodes a methyl cytosine dioxygenase enzyme that promotes DNA demethylation; thus, TET2 exerts its important function on chromatin modification in cells [111]. TET2-disrupted CD19-CART cells display an epigenetic profile as a central memory phenotype, resulting in sustained cancer remission [112]. Moreover, Jain et al. recently revealed that deleting TET2 in CAR-T cells also results in CAR-T cell hyperproliferation, depending on the AP-1 factor BATF3, while the hyperproliferation is antigen-independent [113]. PR domain zinc finger protein 1 (PRDM1) plays a crucial role in epigenetic regulation during T-cell terminal differentiation. PRDM1 disruption by CRISPR/Cas9 promotes CAR-T cells to maintain an early memory phenotype and polyfunctional cytokine secretion under repeated antigen stimulation. PRDM1-disrupted CAR-T cells exhibit enhanced chromatin accessibility of the genes that regulate memory phenotype formation and maintenance, thereby resulting in the generation of early memory T cells, which improves CAR-T cell durability in vivo [114]. DNA methylation modified by DNA methyltransferase (DNMTs) can result in T-cell exhaustion and impair T-cell-based immunotherapy [115]. As a major component during the de novo DNA methylation process, ablating DNMT3A empowers CAR-T cells with resistance to exhaustion and preserves their proliferation potential. Thus, NDMT3A-knockout CAR-T cells display robust anti-tumor efficacy compared to wildtype CAR-T cells [116].

### 3.5. Screening for New Targets in CAR-T Cells for Long-Time Persistence

The current studies mostly focus on knocking out genes with known function by CRISPR/Cas9 in CAR-T cells. These genes have been proven to play crucial roles in regulating CAR-T cell differentiation, survival, and effector function, and previously mentioned studies have obtained remarkable effects in improving the persistence and anti-tumor activity of CAR-T cells. Nevertheless, TME is complex, and it has various inhibitory factors and signals. The discovery of new and undetected genes regulating the CAR-T cell function is necessary for the enhancement of CAR-T cell clinical efficacy. Thus, CRISPR screening is emerging as a method to find the key regulators or genes that perform a specific function or regulate a specific cell phenotype. This strategy utilizes the CRISPR/Cas9 gene engineering system and libraries of sgRNAs rather than one sgRNA, which is able to target every gene in the genome [117]. For the past few years, CRISPR/Cas9-mediated genome-wide screening platforms have been an effective tool for the identification of novel targets for cancer immunotherapy [118]. Here, we summarize the studies using genome-wide CRISPR screening to discover new genes that play important functions in T-cell activation, differentiation, effector function, and exhaustion.

#### 3.5.1. Screen for Exhaustion and Memory Phenotype Targets

The molecular determinants of CAR-T cell exhaustion and differentiation still remain poorly understood. To investigate the underlying mechanisms, a series of studies are conducted to identify new targets and regulators in the regulation of CAR-T cell exhaustion and memory cell formation. Julia et al. conducted different genome-wide CRISPR knockout screens under several distinct immunosuppressive models in order to discover new genes that regulate T-cell anergy. They identified that the expression of RASA2 decreased upon acute TCR activation while gradually increasing under chronic antigen stimulation. RASA2 deficiency improves MAPK signal pathway activation, and RASA2-ablated CAR-T cells reveal increased activation, cytokine production, and metabolic activity and prolong the survival of patients with liquid or solid cancers. This study demonstrates that RASA2 is a novel target to boost both long-term persistence and the effector function of CAR-T cells [119]. Katherine A. et al. conducted two CRISPR knockout screens in human CAR-T cells under chronic antigen exposure and identified MED12 (mediator complex subunit 12) and CCNC (cyclin C) as the primary regulators of the T-cell effector function. MED12 and CCNC are constituent parts of the mediator kinase module. MED12 or CCNC-disrupted CAR-T cells exhibit augmented expansion, cytokine secretion, and tumor killing potency. In addition, MED12 deletion sustains the effector phenotype of CAR-T cells and enhances their IL2RA expression and IL-2 sensitivity. This study identifies that the mediator kinase module is a new regulator for enhancing CAR-T cell effector activity [120]. In addition, Julia A. et al. utilized a complementary in vitro and in vivo screening strategy, including an in vitro exhaustion assay accompanied by a genome-wide CRISPR screen and in vivo follow-up screens. This strategy uncovered a series of epigenetic regulators involved in chromatin remodeling, including the cBAF and INO80 complexes. cBAF perturbation enhances T-cell persistence, prevents exhaustion, and improves tumor control, while INO80 disruption influences gene expression, which plays an important role in metabolic functions. Furthermore, the ablation of the cBAF complex subunit Arid1a significantly prolongs T-cell persistence and anti-tumor ability after adoptive T-cell transfer [121]. Likely, Marcel P. et al. also utilized genome-wide CRISPR screening to identify the new mediators of T-cell exhaustion. They first develop an T cell antigen-specific exhaustion in vitro model and sort exhausted and less exhausted T cells. Based on gRNA NGS sequencing and the individual gene knockout experiments, they identified SNX9 as a potential regulator for T-cell exhaustion. SNX9 disruption decreases PLCγ1, Ca^2+^ and NFATc2-mediated TCR signaling, and NR4A1/3 and TOX expression, which are essential transcription factors in T-cell exhaustion. Meanwhile, SNX9 ablation enhances the memory phenotype and anti-tumor efficacy of CAR-T cells [122]. Zhang et al. also established an ex vivo CAR-T cell anergy model to mimic in vivo CAR-T cell exhaustion and performed CRISPR/Cas9 screening to identify the crucial genes that contribute to the exhaustion of CAR-T cells. They found that BATF-ablated CAR-T cells revealed vigorous anti-tumor ability. Meanwhile, BATF depletion improves CAR-T cell resistance to exhaustion and the central memory phenotype of CAR-T cells [123]. These studies provide new and promising targets to alleviate CAR-T cell exhaustion and prolong their survival.

#### 3.5.2. Screening for Activation, Expansion, and Effector Function Targets

The robust expansion and maintenance of effector function after CAR-T cell infusion are essential for an effective therapeutic outcome. Thus, it is necessary to find new genes that are crucial for CAR-T cell activation, proliferation, and tumor killing ability. Legut et al. performed a genome-wide gain-of-function screening in primary T cells and showed that the overexpression of LTBR could improve CAR-T cell activation and proliferation via the canonical NF-κB pathway [124]. Shang et al. performed a genome-wide CRISPR screening in IL13Rα2-CART cells cocultured with glioblastoma stem cells and identified the novel regulators in the regulation of CAR-T cell effector activity, including TLE4 and IKZF2. TLE4-KO or IKZF2-KO IL13Rα2-CART cells reveal enhanced cytotoxic potency and reduced exhaustion after being cocultured with cancer cells. Single-cell RNA sequencing shows that TLE4-KO or IKZF2-KO IL13Rα2-CART cells upregulate the expression of effector activity-associated genes and downregulate the expression of exhaustion-associated genes. In addition, TLE4 or IKZF2 perturbation also improves the expansion and killing ability of HER2-targeted CART cells. Furthermore, this study simultaneously screens the GBM stem cells that are cocultured with CAR-T cells and uncovers the essential genes that are inducing resistance to CAR-T cells. This reciprocal CRISPR screening enables investigators to identify the genes that are both in CAR-T cells and tumor cells, thereby regulating the efficacy of CAR-T cells [125]. To elucidate the characteristics of therapeutically effective anti-tumor T cells, Devikala et al. selected four phenotypic qualities of effective anti-tumor T cells: cell proliferation, stem cell-like memory, metabolic fitness, and genomic stress. After the selection of phenotypes, the investigators conducted a multitype CRISPR/Cas9 screening on TCR-driven kinases and identified p38 kinase as playing a crucial role in regulating all four phenotypes of T cells. The pharmacological inhibition of p38 enhances CAR-T cell persistence and anti-tumor efficacy [126]. Ye et al. developed a hybrid screening system where the single-guide RNA library and the Sleeping Beauty (SB) transposon are packed in an adeno-associated virus (AAV). This strategy increases gene-editing efficiency. Using the new method, the investigators performed in vivo screens for membrane protein targets in mouse CD8^+^ T cells and uncovered new targets that could improve the CD8^+^ T cell effector function, including Pdia3, Mgat5, and Emp1. In addition, PDIA3 perturbation enhances the tumor killing potency of EGFRvIII-CART cells against glioblastoma [127]. Besides loss-of-function, gain-of-function could also be applied in CRISPR screening and help identify new targets that enhance the anti-tumor potency in CAR-T cells. Ye et al. developed a dead-guide RNA (dgRNA)-based CRISPR activation screening in CD8^+^ T cells and identified novel targets that could be utilized to boost the CAR-T cell effector function. They found that a targeted knock-in or the overexpression of PRODH2 encoding an enzyme in proline metabolism, which is an important metabolic process in T-cell effector activity, enhances the in vivo anti-tumor efficacy of CAR-T cells in multiple cancer models [128]. The targeted knock-in of PRODH2 in CD8^+^ T cells reprograms proline metabolism. Meanwhile, the overexpression of PRODH2 in CAR-T cells enhances their cytotoxic ability in vitro and in vivo, restructures gene expression and metabolism, and improves their long-term anti-tumor activity [129]. The above studies demonstrate that CRISPR screening is an encouraging tool for identifying new targets to enhance CAR-T cell durability and anti-tumor potency.

### 3.6. CRISPR/Cas9 Technology in CAR-T Cells to Conquer Other Limitations

Besides the differentiation status and dysfunction, other factors could also limit the persistence of CAR-T cells. At present, the CAR-T cells applied in the clinic are generated from patients’ autologous T cells. Nevertheless, cancer patients receive multiple rounds of radiotherapy and chemotherapy, leading to a stark reduction in circulating T cells. Meanwhile, the anti-tumor activity of most T cells derived from cancer patients is impaired, making it difficult to sustain effective anti-tumor function after being transformed into CAR-T cells. Thus, off-the-shelf allogeneic CAR-T cells are the ideal choice for immunotherapy in tumors. However, graft-versus-host-disease (GVHD) is a major obstacle that limits the translational application of allogeneic CAR-T cells. GVHD is mainly induced by the recognition of recipient alloantigens by donor TCRs. In addition, the recognition of donor HLA molecules by the recipient’s immune cells results in graft rejection. Thus, the disruption of TCR and HLA-I expression to generate off-the-shelf universal CAR-T cells is necessary. CRISPR/Cas9 is a powerful tool to destroy TCR and HLA-I. Preclinical and clinical study data have demonstrated that the ablation of TCR and/or HLA-I could efficiently reduce GVHD toxicity [130,131,132]. 

Besides rendering CAR-T cell resistant to immunosuppressive cytokines, CRISPR/Cas9 could help CAR-T cells secrete specific cytokines that are beneficial for anti-tumor potency. For example, IL-15 [133] and IL-18 [134] have been revealed to improve the anti-tumor activity and long-term persistence of CAR-T cells. Also, IL-23 could promote granzymeB production, decrease PD-1 expression, and increase CAR T-cell expansion [135]. Thus, utilizing CRISPR/Cas9 to knock-in the specific cytokine could be a promising approach to increase the tumor killing ability and long-term persistence of CAR-T cells.

## 4. Current Clinical Application of CRISPR/Cas9 Technology in CAR-T Cell Therapy

With the gradual maturity and continuous development of CRISPR/Cas9 technology, more and more clinical trials are being conducted to investigate the safety and feasibility of this genome-editing tool for clinical transformation. Most clinical trials are under phase I and are performed to evaluate the safety and efficiency of universal CAR-T cells in hematological malignancies. Meanwhile, there are several trials that are concerned with immune checkpoint-disrupted CAR-T cells. Some clinical trials have produced encouraging results. For instance, in a phase I, open-label, non-randomized clinical trial, Giorgio et al. utilized the CRISPR/Cas9 system to delete the expression of TRAC and CD52 in CD19-CART cells and treat six pediatric patients with relapsed/refractory CD19-positive B-ALL. All the patients received CAR-T cell treatment that manifested different complications, like grade II cytokine release syndrome and grade IV neurotoxicity GVHD in the skin. However, these complications were resolved after appropriate intervention [136]. Another clinical trial was conducted to investigate the role of CRISPR/Cas9-engineered universal CD19/CD22 dual-targeted CART cells in treating patients with r/r B-ALL. The investigators disrupted the TRAC region and CD52 gene in CD19/CD22 dual-targeted CART cells and evaluated their efficacy in r/r B-ALL patients. The clinical trial results demonstrated that 83.3% complete remission was reached on day 28 after engineered CART cell infusion [132]. These studies demonstrate the feasibility and safety of CRISPR-engineered CAR-T cells in hematopoietic malignancies. In addition, Wang et al. generated PD-1 and TCR-disrupted mesothelin-specific CART (MPTK-CART) cells by CRISPR/Cas9 and applied them to patients with MSLN^+^ solid tumors in a phase I study. Dose-limiting toxicity or unexpected adverse events were not observed in any of the 15 patients. Two patients achieved stable disease. However, in mesothelin-positive solid tumors, TCR deficiency impaired the persistence of CAR-T cells. This study demonstrated the feasibility and safety of PD-1-deficient CAR-T cells and revealed the function of TCR for the durability of CAR-T cells in solid tumors [137]. These clinical trial results show that CRISPR-engineered CAR-T cell therapy has a bright future in clinical applications. More detailed clinical trials of CRISPR/Cas9-engineered CAR-T cells are shown in Table 1.

## 5. Challenges of the Application of CRISPR/Cas9 in CAR-T Cells

Several challenges with the CRISPR/Cas9 system limit its application to CAR-T cells. The first challenge is off-target mutations. Because the human cell genome is complex and totally exact complementarity is not required between sgRNAs and their targeted DNA location, the sgRNAs might guide Cas9 proteins to the wrong DNA site because of the similarities within the genome. A mismatch may lead to unintended mutations in the genome and disrupt the function of non-targeted genes [138]. Cuts at off-target sites would generate single-stranded breaks (SSBs) in the DNA and impair the specificity of CRISPR/Cas9 technology [139]. Meanwhile, a mismatch to the undesired DNA locations may induce the dysregulation of protein expression, which leads to a risk of malignancy [140]. Thus, rational designs of sgRNAs by bioinformatics tools to increase their accuracy are an effective approach to avoid off-target toxicity [141]. Meanwhile, different Cas nuclease can also reduce off-target influence. In addition, delivery strategies for the CRISPR/Cas9 system also induce the off-target toxicity. It has been revealed that the RNP delivery system decreases the occurrence of off-target effects due to the limited existence of the CRISPR/Cas9 system in the engineered cells [78]. The second challenge is the Cas9 protein-related immunogenicity in the in vivo CRISPR/Cas9 gene editing [142]. In vivo gene editing delivers the CRISPR/Cas9 system directly into the body rather than infusing CRISPR/Cas9-engineered CAR-T cells. Once the CRISPR/Cas9 system is delivered into the body, the immune system will recognize the Cas9 protein as foreign and develop an immune response against it, which induces fast degradation of the Cas9 protein. This degradation will lead to the failure of gene editing [143]. The immune reaction against Cas9 will largely reduce the efficiency of in vivo gene editing in CAR-T cells. The current strategies to overcome immunogenicity include epitope masking and regulating its expression or inducing degradation of Cas9 protein [144]. The delivery system is another challenge that limits the efficiency and application of CRISPR/Cas9. Different delivery systems, such as viral and non-viral delivery techniques, have been utilized in the CRISPR/Cas9 system. Each approach has its advantages and limitations. Choosing an efficient and safe delivery approach to send the CRISPR/Cas9 system into cells is necessary for the function exerted by CRISPR/Cas9 [145].

## 6. Conclusions

**Figure 1:**
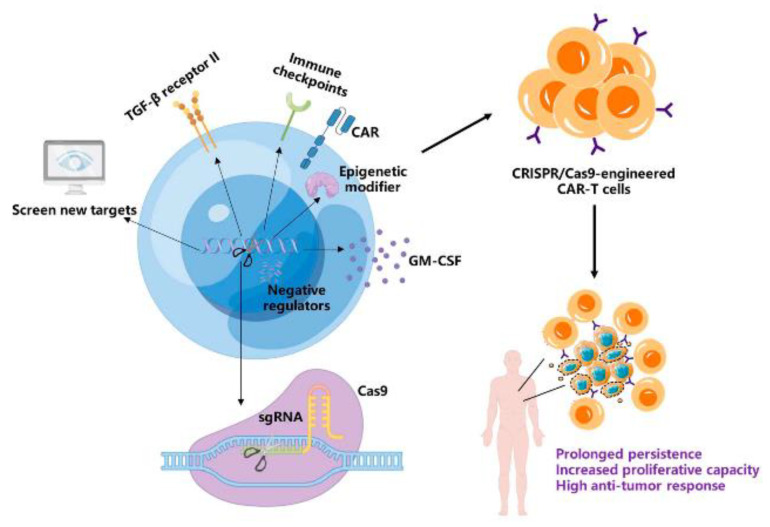
A schematic of applications of CRISPR/Cas9 technology in CAR-T cells to improve long-term persistence (By Figdraw, www.figdraw.com, accessed on 27 June 2023).

Although CAR-T cell therapy has achieved some remarkable results, there are still many factors limiting its wide application in clinical cancer immunotherapy. The poor persistence of CAR-T cells is a dominant challenge that limits long-term cancer remission. The terminally differentiated phenotype and exhausted status play important roles in impairing the survival and persistence of CAR-T cells. Thus, promoting CAR-T cells to maintain the memory phenotype and avoid exhaustion could significantly enhance the anti-tumor activity and long-term persistence of CAR-T cells. The differentiation and exhaustion of CAR-T cells are both regulated by the genome. Hence, genome editing is a promising tool for manipulating specific gene expression to regulate the differentiation process and reduce exhaustion. With high accuracy and efficiency, CRISPR/Cas9 technology has become the most widely used gene-editing tool in cancer immunotherapy. The CRISPR/Cas9 system can knockout or knock-in specific genes that regulate differentiation and exhaustion in CAR-T cells, leading to an improvement in tumor killing and long-term persistence. In addition, CRISPR screens can identify novel targets to improve CAR-T cell anticancer efficacy (Figure 1). However, some challenges in CRISPR/Cas9 technology need to be solved, such as off-target mutations and Cas9 protein immunogenicity. Hence, more profound research on CRISPR/Cas9 needs to be conducted. Meanwhile, large-scale clinical trials for CRISPR/Cas9-engineered CAR-T cells should be performed to evaluate their safety, efficacy, and accessibility. We hope that CRISPR/Cas9-engineered CAR-T cells have a bright future in cancer immunotherapy.

## Figures and Tables

**Table 1:** Registered clinical trials of CRISPR/Cas9-engineered CAR-T cells for cancer therapy.

NCT Number	Phase	Target Gene	CAR-T Cell	Cancer	Location	Status
NCT03545815	I	TRAC and PDCD1	Mesothelin- CART	Mesothelin-positive multiple solid tumors	China	Completed
NCT04557436	I	TRAC and CD52	CD19-CART	Refractory B-cell leukemia	The United Kingdom	Active; not recruiting
NCT03166878	I/II	B2M	CD19-CART	B-cell leukemia	China	Recruiting
NCT04244656	I	TCR and MHC class I	BCMA-CART	Multiple myeloma	The United States	Active, Not Recruiting
NCT04438083	I	TRAC, B2M and CD70	CD70-CART	Relapsed or refractory renal cell carcinoma	The United States	Active; not recruiting
NCT05643742	I/II	TRAC and B2M	CD19-CART	Relapsed or refractory B-cell malignancies	The United States	Recruiting
NCT03747965	I	PDCD1	Mesothelin-CART	Mesothelin-positive multiple solid tumors	China	Completed
NCT04035434	I	TRAC and B2M	CD19-CART	Relapsed or refractory B-cell malignancies	The United States	Recruiting
NCT03166878	I/II	TRAC and B2M	CD19-CART	Relapsed or refractory CD19^+^ leukemia and lymphoma	China	Completed
NCT03398967	I/II	TRAC and CD52	CD19/20- or CD19/22-UCART	Relapsed or refractory hematological malignancies	China	Unknown
NCT04976218	I	TGF-β receptor II	EGFR-CART	Advanced EGFR-positive solid tumors	China	Recruiting
NCT05812326	I/II	PDCD1	MUC1-CART	MUC1-positive advanced breast cancer	China	Completed
NCT04037566	I	HPK1	CD19-CART	Hematopoietic malignancies	China	Recruiting
NCT03203369	I	TCR	CD123-CART	Blastic plasmacytoid dendritic cell neoplasm	The United States	Discontinued
NCT04637763	I	Unknown	CD19-CART	Relapsed/refractory B-cell non-Hodgkin lymphoma	The United States	Recruiting

Data extracted from https://clinicaltrials.gov/ (last accessed 1 June 2023).
